# Postoperative drainage for 6, 12, or 24 h after burr-hole evacuation of chronic subdural hematoma in symptomatic patients (DRAIN-TIME 2): study protocol for a nationwide randomized controlled trial

**DOI:** 10.1186/s13063-022-06150-x

**Published:** 2022-03-14

**Authors:** Mads Hjortdal Grønhøj, Thorbjørn Søren Rønn Jensen, Ann Kathrine Sindby, Rares Miscov, Torben Hundsholt, Birgit Debrabant, Carsten Reidies Bjarkam, Bo Bergholt, Kåre Fugleholm, Frantz Rom Poulsen

**Affiliations:** 1 Department of Neurosurgery, Odense University Hospital, J.B. Winsløwsvej 4, DK-5000 Odense, Denmark; 2 Department of Clinical Research and BRIDGE - Brain Research Inter-Disciplinary Guided Excellence, University of Southern Denmark, DK-5000 Odense, Denmark; 3 Department of Neurosurgery, Rigshospitalet, Blegdamsvej 9, DK-2100 Copenhagen, Denmark; 4 Department of Neurosurgery, Aarhus University Hospital, Palle Juul-Jensens Boulevard 99, DK-8200 Aarhus, Denmark; 5 Department of Neurosurgery, Aalborg University Hospital, Hobrovej 18-22, DK-9000 Aalborg, Denmark; 6 Department of Mathematics and Computer Science, University of Southern Denmark, Campusvej 55, DK-5000 Odense, Denmark; 7 Epidemiology, Biostatistics and Biodemography Department of Public Health, University of Southern Denmark, J.B. Winsløwsvej 9b, DK-5000 Odense, Denmark

**Keywords:** Chronic subdural hematoma, Neurosurgery, Neurology, Drain, Randomized control trial, Recurrent chronic subdural hematoma

## Abstract

**Background:**

Chronic subdural hematoma (CSDH) is a common acute or subacute neurosurgical condition, typically treated by burr-hole evacuation and drainage. Recurrent CSDH occurs in 5–20% of cases and requires reoperation in symptomatic patients, sometimes repeatedly. Postoperative subdural drainage of maximal 48 h is effective in reducing recurrent hematomas. However, the shortest possible drainage time without increasing the recurrence rate is unknown.

**Methods:**

DRAIN-TIME 2 is a Danish multi-center, randomized controlled trial of postoperative drainage time including all four neurosurgical departments in Denmark. Both incapacitated and mentally competent patients are enrolled. Patients older than 18 years, free of other intracranial pathologies or history of previous brain surgery, are recruited at the time of admission or no later than 6 h after surgery. Each patient is randomized to either 6, 12, or 24 h of passive subdural drainage following single burr-hole evacuation of a CSDH. Mentally competent patients are asked to complete the SF-36 questionnaire. The primary endpoint is CSDH recurrence rate at 90 days. Secondary outcome measures include SF-36 at 90 days, length of hospital stay, drain-related complications, and complications related to immobilization and mortality.

**Discussion:**

This multi-center trial will provide evidence regarding the shortest possible drainage time without increasing the recurrence rate. The potential impact of this study is significant as we believe that a shorter drainage period may be associated with fewer drain-related complications, fewer complications related to immobilization, and shorter hospital stays—thus reducing the overall health service burden from this condition. The expected benefits for patients’ lives and health costs will increase as the CSDH patient population grows.

**Trial registration:**

ISRCTN Registry ISRCTN15186366. Registered in December 2020 and updated in October 2021. This protocol was developed in accordance with the SPIRIT Checklist and by use of the structured study protocol template provided by BMC Trials.

**Supplementary Information:**

The online version contains supplementary material available at 10.1186/s13063-022-06150-x.

## Administrative information

**Table:** 

Title {1}	Postoperative drainage for 6, 12, or 24 h after burr-hole evacuation of chronic subdural hematoma in symptomatic patients (DRAIN-TIME 2): study protocol for a nationwide randomized controlled trial
Trial registration {2a and 2b}	ISRCTN15186366. 10.1186/ISRCTN15186366. Registered in December 2020
Protocol version {3}	7th of September 2021, version 2
Funding {4}	This study is funded by the four neurosurgical departments in Denmark. No external funding is involved.
Author details {5a}	^1^Odense University Hospital Dept. of Neurosurgery J.B. Winsløwsvej 4, DK-5000 Odense Denmark E-mail: mads.groenhoej@rsyd.dk ^2^Rigshospitalet Dept. of Neurosurgery Blegdamsvej 9, DK-2100 Copenhagen Denmark E-mail: tjens07@gmail.com ^3^Aarhus University HospitalDept. of Neurosurgery Palle Juul-Jensens Boulevard 99, DK-8200 Aarhus Denmark E-mail: ansind@rm.dk ^4^Aalborg University Hospital Dept. of Neurosurgery Hobrovej 18-22, DK-9000 Aalborg Denmark E-mail: r.miscov@rn.dk ^5^Aalborg University Hospital Dept. of Neurosurgery Hobrovej 18-22, DK-9000 Aalborg Denmark E-mail: tohu@rn.dk ^6^Department of Mathematics and Computer Science University of Southern Denmark Campusvej 55, DK-5000 Odense Denmark E-mail: bdebrabant@sdu.dk ^7^Epidemiology, Biostatistics and Biodemography Department of Public Health University of Southern Denmark J.B. Winsløwsvej 9b, DK-5000 Odense Denmark E-mail: bdebrabant@sdu.dk ^8^Aalborg University Hospital Dept. of Neurosurgery Hobrovej 18-22, DK-9000 Aalborg Denmark E-mail: c.bjarkam@rn.dk ^9^Aarhus University HospitalDept. of Neurosurgery Palle Juul-Jensens Boulevard 99, DK-8200 Aarhus Denmark E-mail: bo.bergholt@rm.dk ^10^Rigshospitalet Dept. of Neurosurgery Blegdamsvej 9, DK-2100 Copenhagen Denmark E-mail: kaare.fugleholm.buch@regionh.dk ^11^Odense University Hospital Dept. of Neurosurgery J.B. Winsløwsvej 4, DK-5000 Odense Denmark E-mail: frantz.r.poulsen@rsyd.dk ^12^Department of Clinical Research and BRIDGE—Brain Research Inter-Disciplinary Guided Excellence, University of Southern Denmark, DK-5000 Odense, Denmark
Name and contact information for the trial sponsor {5b}	Not applicable, no trial sponsor
Role of sponsor {5c}	Not applicable, no study sponsor or funders

## Introduction

### Background and rationale {6a}

Chronic subdural hematoma (CSDH) is one of the most common neurosurgical conditions, especially in older patients, and causes serious morbidity and mortality [1]. It consists of a collection of partly or totally liquefied old blood on the brain’s surface between the dura mater and arachnoid [2]. The exact pathogenesis of CSDH remains unclear, but head trauma is known to be an important factor. CSDH can develop following only a minor injury to the head but also in the absence of a known trauma [1]. Symptoms of CSDH depend upon the size of the hematoma and specific location and may include headache, focal neurological deficits, speech problems, gait disturbance, falls, cognitive decline, confusion, and decreased consciousness. Patients with significant symptoms are admitted to a neurosurgical department for operative treatment. Burr-hole evacuation is the standard procedure in most centers. However, recurrence of CSDH is observed in 5–20% of patients, requiring readmission and further surgery. Although it is still debated whether to use subperiosteal versus subdural drain [3], there is evidence that postoperative drainage is effective in reducing the symptomatic recurrence of CSDH [4]. The shortest possible postoperative drainage period without compromising the drainage effect is preferable, both from a medical and a health economic perspective. It has been shown to be much more advantageous to remove the drain at 48 h compared to 96 h as the rate of morbidities was significantly decreased [5]. Unpublished data from a newly terminated Danish multi-center, randomized controlled trial (DRAIN-TIME 1) of postoperative drainage time could not show 48 h drainage to be superior to 24 h regarding the recurrence rate or death at 3 months.

DRAIN-TIME 2 is a nationwide, multi-center, randomized controlled trial investigating the shortest possible subdural drainage time without significantly increasing the recurrence rate. Patients will be randomized to a postoperative drainage period of 6, 12, or 24 h. The potential impact of this study is significant as we believe that a shorter drainage period may be associated with fewer drain-related complications, fewer complications related to immobilization, and shorter hospital stays.

#### Trial rationale

We hypothesize that a drainage period of 6 and/or 12 h is non-inferior to 24 h of drainage regarding recurrence rate, and we hypothesize that the shortest possible drainage time is associated with fewer drain-related complications, faster mobilization, and shorter hospital stays.

### Objectives {7}

#### Primary objective

The primary objective is to investigate if the drainage period of 6 and**/**or 12 h is non-inferior to 24 h of drainage regarding CSDH recurrence rate at 3 months.

#### Secondary objectives

The following are the secondary objectives:

- Compare the long-term (90 days) outcome measures
- Compare the drain- and immobilization-related complications
- Compare the length of hospital stay (at the department of neurosurgery)

#### Exploratory (mechanistic) objective (sub-study)

The exploratory objective is to assess the composition of proteomics, inflammatory, and angiogenetic markers in the blood, CSDH fluid, surrounding membranous tissue, and draining fluid.

### Trial design {8}

The DRAIN-TIME 2 study is a Danish multi-center, national randomized controlled non-inferiority trial with three parallel arms. The arms correspond to 6, 12, and 24 h of drainage, where the former two arms are experimental and the 24 h group is the common control arm. Online randomization with a 1:1:1 allocation will be performed.

The trial uses a multi-arm, multi-stage (MAMS) design, which enables adaptive reductions of the number of experimental arms considered during the trial, with the aim to lower the required sample size on average. The applied design is described in Bratton et al. [6]. Our trial is organized into five stages. After each stage, non-inferiority of both arms is tested during an interim analysis at stage-specific significance levels. Significant arms continue to the next stage, whereas non-significant arms can be dropped (non-binding).

The final non-inferiority test decision is taken after the final stage (or as soon as both experimental arms are stopped) and uses all available data. Interim and final analyses rely on the same primary outcome and the same non-inferiority margin.

## Methods: participants, interventions, and outcomes

### Study setting {9}

This study is performed by the Danish Chronic Subdural Hematoma Study group (DACSUHS), which is a national steering committee with participants from all neurosurgical departments in Denmark. DACSUHS coordinates CSDH management and research in Denmark. All study sites are thus in Denmark, and all Danish neurosurgical departments are participating. There are no private facilities for cranial neurosurgery in Denmark. Patients are admitted to their local neurosurgical department after a computed tomography (CT) has confirmed the diagnosis. Local clinical neurosurgical teams review the patients upon admission and will assess the eligibility for the DRAIN-TIME 2 study. The decision for surgery is made on an individual basis by the neurosurgical teams together with the patient (if possible) and their family. The study was approved on two levels by the Danish Regional Committees on Health Research Ethics, so both incapacitated and mentally competent patients can be enrolled.

### Handling anti-coagulant therapy

Preoperative management (pause) of anti-coagulant therapy was performed in accordance with the recommendations of the Danish Society for Hemostasis and Thrombosis, published in 2016 (https://www.dsth.dk/pdf/PRAB_2016_WEB.pdf). There is no solid evidence regarding the time of restart of anti-coagulant treatment post-surgery, in order to minimize the risk of recurrence on the one hand and prevent thromboembolic complications on the other hand. Therefore, DACSUHS made a consensus decision regarding the restart of treatment: Anti-coagulant medication can be resumed 14 days after surgery (7 days if patients receive low-molecular-weight heparin, as recommended by the Danish Society for Hemostasis and Thrombosis for certain high-risk patient categories). All patients who receive anti-coagulant treatment for atrial fibrillation are referred to the department of cardiology at the time of discharge, in order to determine whether they are candidates for atrial ablation, so that anti-coagulant therapy can be avoided.

### Public and/or patient involvement in designing the study protocol

The study protocol was designed by DACSUHS. This study builds on the results from the DRAIN-TIME 1 study, solely focusing on drainage time post-surgery. All other patient care is the standard treatment. Therefore, we have considered any public and/or patient involvement unfruitful in the process of designing the present study protocol.

### Eligibility criteria {10}

Screening of patients to determine eligibility for participation in the study will be performed by the neurosurgical team upon admission according to the following criteria.

#### Inclusion criteria

The following are the inclusion criteria:

- Adult patients (≥ 18 years).
- Minimum 2 weeks’ time span from known head trauma.
- Patients with symptomatic CSDH confirmed on CT or magnetic resonance imaging (MRI), admitted to a neurosurgical department for operative treatment.
- Patients undergoing a single burr-hole evacuation and placement of a passive subdural drain.
- All patients independent of GCS at admission can be included.

#### Exclusion criteria

The following are the exclusion criteria:

- Patients with known abnormalities in their cerebrospinal fluid (protein and glucose levels, cell count, and type)
- Patients with changes or abnormalities in their normal cerebrospinal fluid dynamics, e.g., obstructive hydrocephalus, normal pressure hydrocephalus, intracranial hypotension, and ventricular peritoneal shunt
- Patients with additional/previously intracranial pathology that requires/has required neurosurgical treatment (e.g., brain tumor, vascular malformation, abscess)
- Patients with recurrent CSDH or with previous craniotomy or other transcranial surgery (for any reason)

### Who will take informed consent? {26a}

Informed consent will be obtained by a member of the research team upon admission or immediately after surgery. The Danish confidentiality law requires that the patient and any relatives are orally informed about the trial, including its rationale and overall purpose. Written patient information and the consent form will also be handed out and reviewed together with the patient. Both oral and written consent must be obtained from the patient before study randomization. The patients will be made aware of the right to a reflection period and that they can withdraw their consent at any stage. Patients who do not wish to take part in the trial will have a postoperative drainage period of 24 h, which is the current standard treatment in Denmark.

At Odense University Hospital and Rigshospitalet, we will perform an exploratory/mechanistic sub-study. Patients will be given additional patient information and an additional consent form so that they can consider whether they would like to take part in the sub-study.

Patients who are temporarily incapacitated due to severe symptoms can still be enrolled in the trial if oral and written consent is obtained from both A and B (see below) no later than 6 h post-surgery:

A) The patient’s legal representative. If at any stage the patient’s legal representative chooses to withhold or withdraw the consent, the patient will be excluded from the trial.
B) A healthcare professional (medical doctor) who is independent of the interests of the persons responsible for the trial and of interests in the project in general. The consent may initially be oral, and the doctor’s first and last name must be noted in the medical record.

The patient can then be randomized.

Mentally incapacitated patients relinquish the authority, that is the competent patients’ right, to choose among professionally acceptable alternative treatments or to participate in research projects. While standards to determine intellectual capacity remain unclear, a practical approach is to demonstrate that a patient is able to describe the physician’s view of the situation and to understand the physician’s opinion as to the best intervention [7]. We consider patients to be incapacitated if they are unable to participate in the medical examination; are not aware of the time, place, and personal information; or are somnolent or comatose.

Patients who regain capacity post-surgery will be informed about the clinical trial, and consent will be sought. In most cases, this will happen during the stay at the department of neurosurgery. If this is not the case, evaluation of capacity will be performed at 90 days follow-up.

All patients admitted to the neurosurgical departments will receive standard care and will be monitored pre- and postoperatively as per routine clinical practice. Patients will either be discharged to home or transferred to another department or hospital. The Short-Form 36 (SF-36) questionnaire will be used for the 90 days follow-up and will be handed out to patients before discharge. In addition, the patient will receive the questionnaire by mail after 90 days. A member of the research team will contact the patient or the patient’s legal representative by telephone in order to determine their capacity, obtain a Modified Rankin Scale score, and guide the patient through the SF-36 questionnaire. If the patient is considered to be incapacitated, then the patient will be deemed as lost to follow-up concerning SF-36.

### Additional consent provisions for collection and use of participant data and biological specimens {26b}

Not applicable.

### Interventions

#### Explanation for the choice of comparators {6b}

Not applicable. A control/placebo group is not a part of the study design.

#### Intervention description {11a}

This trial follows Danish standard clinical care and treatment published by DACSUHS [8]. Placement of a subdural drain is the standard treatment, and the only deviation is the drainage time. Blood samples are obtained routinely at admission.

In the sub-study, extra blood will be collected and stored, and CSDH fluid and the surrounding membrane will be removed during surgery. This biological material will be collected and stored for later analyses together with fluid collected from the drain during the postoperative period. The results from the sub-study will be published separately.

#### Criteria for discontinuing or modifying allocated interventions {11b}

Discontinuing of the study after randomization can occur in the following situations:

- Signs of infection around the drain tube
- Pronounced leakage from the drain canal
- Patient and/or relative (in case of incapacitated patients) request to withdraw from the study
- Deterioration of the condition which thereby requires repeat surgery or craniotomy during primary hospitalization

#### Strategies to improve adherence to interventions {11c}

The surgeon will state in the medical record the time at which the envelope with the randomization result must be opened (6 h after surgery for all patients). This time will also be stated on the board by the patient’s bed. If the drain is to be removed after 12 or 24 h, the exact time of drain removal is written in the medical journal and on the board after the opening of the randomization envelope at the 6 h time point. A member of the research team will continuously monitor whether this process is carried out correctly.

#### Relevant concomitant care permitted or prohibited during the trial {11d}

All patients receive a standard operation and standard postoperative care. No specific care or interventions are prohibited.

#### Provisions for post-trial care {30}

Neither provisions for ancillary and post-trial care (if any) nor compensation to those who suffer harm from trial participation (if any) are provided by the neurological departments or the DACSUHS consortium. All care and help needed after discharge is provided by the Danish Health Authorities free of charge.

Everyone who receives treatment or purchases medicine in Denmark is covered by the Patient Compensation Association and can file a claim for an injury sustained as a result of the treatment or medication. This also covers participation in clinical trials.

### Outcomes {12}

#### Primary outcome measure

The primary outcome measure is the recurrence rate of chronic subdural hematoma at 3 months postoperatively.

#### Secondary outcome measures

The following are the secondary outcome measures:

- Mortality rate at 90 days.
- Cause of death (e.g., related to recurrence, a symptomatic complication post-surgery comorbidities).
- Modified Rankin Scale at 90 days (Table 1).
- Patient-reported health status assessed by the SF-36 questionnaire (during admission and at 90 days follow-up).
- Length of hospital stay (at the department of neurosurgery before discharge to home or transfer to another hospital).
- Complications related to the drain such as bleeding from the skin, pain, general discomfort, and infection will be monitored as long as the patients are admitted to the neurosurgical department, and therefore, the observation period can vary. Drain-related complications are not to be graded regarding severity, but as “yes or no.” Time (hours) of the onset of symptoms post-surgery will also be noted.
- Complications related to immobilization such as deep venous thrombosis and/or pulmonary embolism and brain infarction will be monitored as long as the patients are admitted to the neurosurgical department, and therefore, the observation period can vary. These complications will be noted as “symptomatic” or “asymptomatic.” The time (hours) of the onset of symptoms post-surgery will also be noted.
- Sub-analyses of patients with recurrence at 90 days: co-morbidities, medications, age, gender, and evaluation of hematoma subtypes on CT from admission (homogenous, separated, mixed, or membranous [2]).

**Table 1:** Modified Rankin Scale (mRS)

mRS score	Description
0	No symptoms
1	No significant disability despite symptoms; able to carry out all usual duties and activities
2	Slight disability; unable to carry out all previous activities but able to look after own affairs without assistance
3	Moderate disability: requiring some help (e.g., with shopping/managing affairs) but able to walk without assistance
4	Moderately severe disability: unable to walk without assistance and unable to attend to own bodily needs without assistance
5	Severe disability; bedridden, incontinent, and requiring constant nursing care and attention
6	Dead

#### Exploratory (mechanistic) outcome measure (sub-study)

To explore possible mechanisms and thereby potential therapeutic targets for reducing recurrence risk, the peripheral and central (local) inflammatory responses will be assessed by collecting blood samples, CSDH fluid, and a biopsy of the surrounding membranous tissue during surgery together with CSDH fluid from the postoperative draining period. Proteomic analysis and inflammatory marker analysis of the collected material will serve as a descriptive study; the results will be compared between recurrent and non-recurrent patients to assess whether the differences in inflammatory mechanisms play a role in the development of recurrent CSDH. The following activities will be conducted:

- Assess the peripheral (blood) inflammatory response in CSDH patients
- Assess the composition of proteomics and inflammatory and angiogenetic markers in the CSDH fluid from surgery and in the fluid obtained during the draining period
- To do immunohistochemical analyses of inflammatory markers on membranous tissue removed during surgery
- Compare fluid composition with hematoma subtype
- Compare fluid composition between recurrent and non-recurrent patients.

The results from this exploratory sub-study will be presented in a separate paper.

#### Participant timeline {13}

As part of the routine standard care at hospital admission, all patients will have a medical history taken and a clinical examination. Table 2 shows a full schedule of trial assessments as per the SPIRIT guidelines.

**Table 2:** Schedule of the trial enrollment, interventions, and assessments

	Enrolment	Surgery	Post-surgery		
Time point	*Ad*	*IO*	*D1*	*D2*	*3 mon*
**Enrollment**					
**Eligibility screen**	X				
**Informed consent**	X				
***Randomization***	X				
**Interventions**					
***Placement of a subdural drain (6, 12, or 24 h)***		X			
**Assessments**					
***Routine labs***	X		X		
***GCS***	X		X		
***mRS***	X		X		X
***Clinical examination***	X				
***SF-36***	X		X^#^		X
***Economic data***					X
CSDH fluid*		X			
CSDH membrane*		X			
Blood*	X				

Schedule of assessments

*Ad* admission, *IO* intraoperative, *D* day, *mon* months, *GCS* Glasgow Coma Scale, *mRS* Modified Rankin Scale, *SF-36* Short Form (36) Health Survey

^*^Only collected in patients recruited to sub-study at Odense University Hospital and Rigshospitalet

^#^If not done the day of admission

#### Sample size {14}

Calculations were based on the primary outcome measure recurrence.

##### Choice of non-inferiority margin

The non-inferiority margin was chosen by the fixed-margin method [9] that is recommended by the United States Food and Drug Administration (FDA) [10]. A historical estimate for the effect of the active control (24 h drainage) compared to no drainage was derived from the meta-analysis from Liu et al. [11]. This publication summarized different surgical procedures including a comparison of drainage vs. no drainage after burr-hole evacuation; drainage times varied between the studies but were typically 48 h.

As the meta-analysis by Liu et al. [11] reports effect sizes as odds ratios (OR) but our sample size calculations required risk differences (RD), we reanalyzed the data used by Liu et al. [11]. This was done as individual participant data analysis using a logistic regression model with cluster robust standard errors. To transform ORs into RDs, we used the multivariate delta method and obtained an approximate 95% confidence interval for either a 7% or 17% difference in the recurrence risk between no drainage and drainage. Consequently, 7% was chosen as the non-inferiority margin.

##### Sample size calculations

The MAMS design required the choice of a number of stages together with the choice of stage-specific decreasing significance levels together with stage-specific power levels, which result in an overall power and a familywise error rate. To inform this decision, we calculated several different designs considering two up to five stages. Stage-specific design parameters were in a try-out manner picked such that the overall power and FWER were approximately equal to 0.8 and 0.028, respectively. Especially, stage-specific significance levels for the initial stage of 0.5, 0.3, and 0.2 were considered. The final design choice was obtained from balancing the resulting expected sample sizes (for different numbers of non-inferior treatment arms) with a greater weight on the sample size in absence of non-inferior treatment arms. This resulted in a final design with five stages and decreasing stage-specific significance levels of alpha = 0.5, 0.3, 0.2, 0.1, and 0.025 together with stage-specific power levels of 0.9 for the first three stages and 0.95 for the last two stages. Stage parameters were chosen such that the overall power for the final comparison of each experimental arm (6 h or 12 h drainage) to control (24 h drainage) is approximately equal to 0.8. The familywise error rate corresponding to the design is approximately 0.028.

Assuming a recurrence rate of 16.3% on the basis of previous DACSUHS studies in all treatment arms (both experimental and control), equal allocation to all treatment arms, and a non-inferiority margin of 7%, we obtained the following cumulative sample sizes.

**Table:** 

	Stage 1	Stage 2	Stage 3	Stage 4	Stage 5
Cumulative sample size per treatment arm reaching the respective stage	91	182	344	477	724
New samples per continued treatment arm and per stage	91	91	162	133	247
Overall cumulative sample size if no arm is dropped in any stage	273	546	1032	1431	2172

After each stage, an interim analysis will be done where non-inferiority of both arms is tested at the chosen stage-specific significance levels. Significant arms continue to the next stage, whereas non-significant arms can be dropped (non-binding). Together with the chosen stage-specific significance levels, the overall pairwise type 1 error rate (for the comparison of one experimental arm to control) equals 0.025 if stopping guidelines (i.e., for dropping of arms before the final stage) are ignored. If stopping guidelines are followed, 0.025 is an upper bound for the overall pairwise type 1 error rate.

The expected sample size for the design if all arms have an identical recurrence rate of 16.3% (implying non-inferiority of both experimental arms) and stopping guidelines are followed is 1928. If both experimental arms are inferior (with recurrence rates exceeding 16.3% by at least 7%, i.e., the non-inferiority margin) and stopping guidelines are respected, the expected sample size is 690.

Sample size calculations were done in STATA17.0 using nstagebin [6, 12]. The corresponding software output is shown in supplementary material A. Reanalysis of the meta-analysis was done in R [13] together with the package miceadds [14].

### Recruitment {15}

Expectations for the recruitment rate are based on the data from the first multicenter national study conducted by DACSUHS (DRAIN-TIME 1, ISRCTN 17021467) that terminated in April 2020. In this study, only patients who were able to give informed consent were enrolled. A total of 37 patients with CSDH were operated on per month in Denmark, and the study recruitment rate was 21 patients per month. It took 20 months to include the total number of 420 patients in DRAIN-TIME 1 (two arms). In contrast to DRAIN TIME 1, both capable and incapacitated patients are enrolled in this DRAIN-TIME 2 study. Depending on whether one of the drainage groups is excluded as a result of the interim analysis, the inclusion period will most likely be 2–4 years.

#### Assignment of interventions: allocation

##### Sequence generation {16a}

Participants will be randomly assigned to one of the three draining groups no later than 6 h postoperatively. Online randomization with a 1:1:1 allocation rate will be performed in each center using the REDCap randomize module in the REDCap database. Randomization is done separately in each center.

##### Concealment mechanism {16b}

Participants will be randomized using REDCap as described above. Allocation concealment will be ensured as the REDCap system will not release the randomization code until the patient has been recruited into the trial. This takes place after the medical history is taken and the clinical examination is performed. The drainage time group randomization is first released after the patient’s data and the signed consent form are uploaded to the database.

##### Implementation {16c}

All patients who give consent to participate and who fulfill the inclusion criteria will be randomized. Randomization will be performed by the on-call neurosurgeon who has operated on the patient and no later than 6 h postoperatively. The randomization result will be placed in a closed envelope.

Both in the medical record and on the envelope, the surgeon will state the time at which the envelope must be opened (6 h after the end of surgery for all patients). Identical information will be stated on the board by the patient’s bed. If the drain is to be removed after 12 or 24 h, the exact time of drain removal is written in the medical journal and on the board after the opening of the envelope (after 6 h of drainage). A member of the research team will continuously monitor whether this process is carried out correctly.

#### Assignment of interventions: blinding

##### Who will be blinded {17a}

Except for the on-call neurosurgeon who operates on the patient, everyone will be blinded to the randomization until 6 h post-surgery. The envelope will be opened by the nurse at exactly 6 h after surgery to determine whether the drain should be removed at that time or after 12 or 24 h. It is a passive drain, and the production rate cannot be affected by any external circumstances, which is why unblinding after 6 h is not considered an issue.

##### Procedure for unblinding if needed {17b}

There are no circumstances where emergency unblinding would be absolutely essential for further management of the patient. Any clinical deterioration or need for repeat surgery would terminate the patient’s participation in the study.

### Data collection and management

#### Plans for assessment and collection of outcomes {18a}

##### Primary outcome

The overall primary clinical outcome is the recurrence of CSDH within 90 days after the initial surgery and drainage. Recurrence is defined as neurological deterioration leading to hospitalization, confirmed recurrent CSDH on cranial imaging (e.g., CT/MRI), and a need for repeat surgery. A retrospective review of the electronic medical record will be used to clarify whether the patient has experienced a recurrence.

Recurrence or no recurrence will be registered in REDCap by a member of the research team after 90 days.

##### Secondary outcomes

A retrospective review of the electronic medical record will be used to clarify whether the patient has died during the observation period, and if so, for what reason.

The Modified Rankin Scale (mRS) is a clinician-reported measure of the patient’s degree of disability. It is widely applied for evaluating the outcome for stroke patients and as an endpoint in most randomized clinical trials [15]. It is a 7-level scale covering the entire range of functional outcomes from no symptoms to death. There is extensive evidence for the validity of the mRS, and the mRS categories correlate with the functional outcomes within the spectrum of stroke pathologies [16]. The limitations of the mRS are the subjective assignment of categories and the reproducibility of the score by examiners and patients [17]. A systematic review and meta-analysis of studies describing interobserver variability of the mRS reported pooled reliability (across 10 studies, *n* = 587 patients) of *κ* = 0.46 and a weighted *κ* of 0.90 [18]. To reduce interobserver variability, all members of the research group will be certified in the use of the mRS (http://rankinscale.org/).

The Short Form-36 Health Survey is a validated, 36-item, patient-reported measure of health-related quality of life (HRQOL) that has been used in a wide spectrum of medical conditions [19–21]. It is one of the most extensively used tools to measure health-related quality of life. It covers eight dimensions of HRQOL (physical functioning, role-physical, bodily pain, general health, vitality, social functioning, role-emotional, and mental health) and has an item on general health perception. Dimension scores can be summed together with differing weightings to generate two summary scores—a physical component score and a mental component score. Higher scores indicate higher HRQOL, and the SF-36 scores range from 0 (worst) to 100 (best) [21]. In the DRAIN-TIME 2 study, the first SF-36 is completed during the initial hospital stay with assistance from a member of the research group. The questionnaire is completed electronically in the REDCap database, via a computer or tablet (Ipad). A second SF-36 questionnaire is given to the patient at discharge and is also sent by secure electronic post (E-boks) to the patient 85 days after surgery. A member of the research group will ring the patient and assist with the completion of this questionnaire after 3 months (approx. 90 days). The responses are entered directly into the online REDCap database.

Using the electronic medical record system, we will collect several data points of interest throughout the study period: number of hours post-surgery before the patient is mobilized and length of hospital stay (i.e., at the department of neurosurgery before discharge to home or transfer to another hospital).

The following will be assessed by direct daily observation during admission to the neurosurgical department: complications related to immobilization (deep venous thrombosis and/or pulmonary embolism, brain infarction) and drain-related complications such as bleeding from the skin, pain, general discomfort, and infection.

#### Plans to promote participant retention and complete follow-up {18b}

The time to follow-up is 90 days, which we believe is a sufficiently short follow-up period to minimize patient attrition and maximize the completeness of data collection. Most symptomatic recurrent CSDH cases related to the primary operation are expected to occur within this time frame [22]. The primary outcome data (CSDH recurrence at 90 days) will be obtained through the electronic medical record. To limit participant burden related to follow-up visits, we will contact each participant by phone after 90 days. Here, we will assist the patient in completing the SF-36 questionnaire (which they will have received via e-mail/E-boks) and determine the mRS category. If a member of the research team assesses that it is not possible to obtain a valid assessment over the telephone, the patient and their relatives are offered attendance at the outpatient clinic.

#### Data management {19}

All patients admitted to one of the neurosurgical departments with a CSDH will be registered in REDCap, which is a worldwide online system developed specifically for non-commercial clinical research. REDCap is administered by the Open Patient data Explorative Network (OPEN) at Odense University Hospital, Odense, Denmark. The data entered will be stored on secure servers in the Region of Southern Denmark. Data are entered via an encrypted connection, are anonymized, and fulfill the demands for data protection. All data entries and changes are logged in REDCap and meet the Good Clinical Practice (GCP) requirements for the use of the electronic case report form (eCRF) in medical trials. The members of the research team are responsible for all data entry. Baseline data obtained from the medical record are registered at admission. After patient consent, online randomization is performed in REDCap. The database is updated when the patient is discharged. At 3 months follow-up, an electronic copy of the SF-36 questionnaire is automatically distributed via E-boks, which is an online secure digital mailbox linked to the patient’s personal Danish registration number.

#### Confidentiality {27}

All study-related information will be stored securely at the study site. All participant information will be stored in locked filing cabinets in areas with limited access. All laboratory specimens, data collection, and administrative forms will be identified by a coded ID [*identification*] number to maintain participant confidentiality. All records that contain names or other personal identifiers, such as informed consent forms, will be stored separately from study records identified by a code number. The electronic database (REDCap) is secured with password-protected access. Forms, lists, and any other listings that link participant ID numbers to other identifying information will be stored in a separate, locked filing cabinet in an area with limited access. Access to the electronic database (REDCap) and to the locked cabinets in areas with limited access is reserved exclusively to members of the national steering committee, DACSUHS. Data processing and statistical work will be performed by exporting the data from REDCap to a secure server. Participant confidentiality will always be maintained.

#### Plans for collection, laboratory evaluation, and storage of biological specimens for genetic or molecular analysis in this trial/future use {33}

A sub-study at Odense University Hospital and Rigshospitalet will be carried out. The biological specimens from this sub-study will be collected and stored in a biobank.

#### Overall objectives and background

Regardless of the choice of surgical procedure, there is a risk of spontaneous recovery/recurrence of the hematoma. Patients who develop recurrence experience significantly greater morbidity and mortality than patients who are cured after the first operation. In recent years, there has been an increasing interest in the composition of the hematoma fluid and in the systemic response as it is likely that the causes of hematoma expansion and recurrence will be found here. Therefore, we will collect hematoma fluid and hematoma membrane during the operation and will collect fluid from the drainage bag when the drain is removed after 6, 12, or 24 h. Furthermore, a blood sample before and after the operation will be obtained for the investigation of systemic changes. The biological material will be used for the analysis of pro- and anti-inflammatory as well as vascular (blood vessel-associated) proteins and peptides. A study of specific neurons and glia (markers of brain impact) will also be performed.

We hope that the study results will shed light on some of the crucial cerebral and systemic processes behind hematoma expansion and whether elimination (leaching) of particular proteins and peptides during surgery influences recurrence risk and postoperative neurological status. Finally, we hope to identify markers that can predict the risk of recurrence.

##### Plasma—collection procedures (methods)

A blood sample will be routinely taken at the time of admission and again on the first postoperative day. At the same time, an additional 10 ml of blood will be collected and distributed in five Sarstedt tubes (2× serum, 1× EDTA-coated, 1× citrate-coated, and 1× buffy-coated). The blood samples will be handled at the respective Departments of Clinical Biochemistry and Pharmacology, where pipetting, freezing, and storage will take place.

##### Chronic subdural hematoma fluid—collection procedures (methods)

Hematoma fluid will be collected from the subdural cavity using a 10-ml syringe. In the operating room, the fluid will be distributed into one Nunc tube of 3.6 ml and two Sarstedt EDTA tubes of 2 ml.

Postoperatively, hematoma fluid from the drainage bag will be retrieved using a 10-ml syringe. Bedside, the fluid will be distributed into one Nunc tube of 3.6 ml and two Sarstedt EDTA tubes of 2 ml.

The material will be picked up by relevant staff and transferred to the respective Departments of Clinical Biochemistry and Pharmacology, where further handling, freezing, and storage will take place.

##### Chronic subdural hematoma membrane—collection procedures (methods)

The hematoma membrane from the subdural cavity will be removed/biopsied (if possible) using appropriate instruments and divided so that half the tissue is fixed in formalin (buffered formaldehyde solution 4%) and the other half is frozen without fixation (− 80°).

All above samples will be logged in, and aliquots will be bar-coded with a unique storage ID generated by the REDCap system. The scientists who carry out the analyses on these materials will not have access to the personal identifiers and will not be able to link the results of these tests to personal identifier information. No individual results will be presented in publications or other reports. Participants will not be informed on an individual basis of any results from these studies.

## Statistical methods

### Statistical methods for primary and secondary outcomes {20a}

#### Primary outcome

Recurrence will be treated as a binary outcome (instead of a time-to-event outcome) due to the time to recurrence being of minor clinical interest compared to the occurrence of the event itself which is of major clinical relevance. As an effect measure, we consider relative risks due to their natural interpretability.

We will use relative risk regression with treatment assignment (6 h vs. 12 h vs. 24 h drainage time) as a categorical covariate and take the different trial centers into account. Adjusted risk ratios with 95% confidence intervals for recurrence rate will be reported. We investigate a non-inferiority hypothesis with a non-inferiority margin of 7% (see the “Sample size {14}” section). We will make two comparisons—one for each experimental arm (6 and 12 h) versus the common control arm (24 h) using likelihood ratio tests. We will use a one-sided test together with stage-specific significance thresholds equaling 0.5, 0.3, 0.2, 0.1, and 0.025 for each of the five stages.

#### Secondary outcomes

Mortality at 3 months will be analyzed by relative risk regression as with the primary outcome. SF-36, mRS, hours until mobilization, and length of hospital stay will be analyzed by linear or ordinal regression. The occurrence of different types of complications among the three study groups will be analyzed with a log-linear Poisson regression model.

For all secondary outcome measures, we investigate the superiority of each of the two experimental arms compared to the active control arm at the final analysis.

We use two-sided tests with a significance threshold of 0.05.

The data analysis will be an intention-to-treat analysis.

### Interim analyses {21b}

Interim analyses of the primary outcome will be conducted at the end of each stage (see the “Sample size {14}” section). The early dropping of an experimental arm (6 or 12 h of drainage) can be chosen if stage-specific significance is not achieved by the respective arm. Secondary outcome measures will only be considered during the final analysis.

### Methods for additional analyses (e.g., subgroup analyses) {20b}

Patients with CSDH recurrence at 90 days will be considered separately. Descriptive tables of CT-based hematoma subtypes, frequency of comorbidities, medications, gender, and descriptive summaries of patient age will be created.

### Methods in analysis to handle protocol non-adherence and any statistical methods to handle missing data {20c}

Loss to follow-up is expected to be rare, and analyses will be done as complete case analyses. All outcomes will be analyzed using intention-to-treat analyses. For the primary outcome, this will be supplemented by an as-treated sensitivity analysis in which the observed drainage times will be used as a continuous covariate instead of the randomly assigned drainage times.

### Plans to give access to the full protocol, participant-level data, and statistical code {31c}

We have no plans for granting public access to the protocol, dataset, or statistical code.

### Oversight and monitoring

#### Composition of the coordinating center and trial steering committee {5d}

**Principal investigator:**

- Preparation of protocol and revisions
- Preparation of written patient information
- Applying for ethical approval
- Organizing steering committee meetings
- Publication of study reports
- Member of steering committee DACSUHS

**Steering committee** (see title page for all members):

- Agreement of final protocol.
- All investigators will be steering committee members; one lead investigator per department will be nominated as the local coordinator.
- Recruitment of patients.
- Reviewing the progress of the study and, if necessary, agreeing on changes to the protocol.
- Data verification.
- Randomization.

**Data manager:**

- External, independent person from OPEN
- Maintenance of the trial IT system (REDCap)
- Monitoring of all activity in the REDCap system
- Data verification

#### Person responsible for data monitoring

An external, independent biostatistician will perform all the statistical analyses throughout the study.

#### Composition of the data monitoring committee, its role, and reporting structure {21a}

A data monitoring committee (DMC) is not established. The decision was because of a relatively simple study design and with potentially minimal risk. The study has a well-described statistical plan that also describes when the interim analyzes should take place and that early dropping of an experimental arm can be chosen if stage-specific significance is not achieved by the respective arm.

However, an external, independent biostatistician will monitor the study data and will be responsible for all the statistical work. The biostatistician is blinded and independent of the study organizers. During the period of recruitment to the study, interim analyses will be supplied together with any other analyses that the research steering committee (DACSUHS) may request. In the light of these interim analyses (three interim analyses and one final analysis), the biostatistician will advise the steering committee based on the previously defined stopping guidelines.

#### Adverse event reporting and harms {22}

An interim analysis is performed as illustrated in the “Sample size {14}” section. If one of the arms is associated with a higher recurrence rate, death, or any other unexpected adverse effect, the steering committee will decide what precautions are to be made.

Otherwise, the operations and postoperative care and handling all follow standard procedures.

#### Frequency and plans for auditing trial conduct {23}

The steering committee will meet 1 month after the trial initiation to evaluate all the aspects related to inclusion and data collection during the hospital stay. The committee will be in constant dialog throughout the study period. Planned meetings will take place after each interim analysis.

#### Plans for communicating important protocol amendments to relevant parties (e.g., trial participants, ethical committees) {25}

No changes will be made in the present study protocol.

#### Dissemination plans {31a}

A final trial report will be written for publication, and trial results will be presented at international meetings.

## Discussion

The recurrence of CSDH is a major challenge, and the driving force behind it is not fully understood. There is solid evidence that postoperative drainage is effective in reducing symptomatic recurrence of CSDH. Despite several studies to date, there is no medical treatment that can limit the risk of recurrence. The optimal drainage period is currently unknown, but from previous studies, we know that drainage more than 48 h is unfavorable. In 2020, DACSUHS completed the DRAIN-TIME 1 study. The study is currently under review, but we report that a drainage time of 24 h has a lower, but non-significant recurrence rate 3 months postoperatively compared to 48 h. Thus, we now recommend 24 h of drainage as the “gold standard” when using a passive subdural drain after burr-hole evacuation.

We designed the DRAIN-TIME 2 study to clarify whether a shorter drainage period (6 or 12 h) is non-inferior to 24 h of drainage regarding recurrence rate. Furthermore, we have included functional outcome measures (e.g., mRS) so that the overall effect on the quality of life can be elucidated. We also investigate the patient-reported quality of life (using the SF-36) in the period prior to and after surgery. Finally, the economic impact of a shorter drainage period will be investigated. In general, neurosurgical departments are smaller, highly specialized departments with a limited number of beds and thus depend on a high turnover of patients. Therefore, the shortest possible postoperative drainage period without compromising the drainage effect is preferable from medical, logistical, and health economic perspectives.

The strengths of this study are that it includes the whole country (all neurosurgical departments in Denmark) and that all patients older than 18 years can be included, including incapacitated patients. The latter group is poorly studied, often limited by the lack of ethical permission, but is particularly interesting as they are the patients who are often in the worst medical condition. We can only speculate whether this group of patients will differ from the others in terms of the effect of the three drainage arms. As this study is conducted in Denmark only, it can also be questioned how applicable the results will be to other populations. However, looking at European Statistics 2020 (EUROSTAT) regarding the population structure and aging, Denmark is “average” compared to the other European countries, and therefore, we believe the results will still be widely applicable [23].

## Trial status

Recruitment commenced on March 1, 2021, and is ongoing under protocol version number 2, with 136 patients recruited as of September 7, 2021. Termination of the study depends on whether one of the treatment arms will be excluded after the interim analyses. Termination is expected in the period from late 2022 to late 2023.

## Protocol amendments

The present manuscript represents protocol version 2, which is the final version under which the trial inclusion was initiated. Protocol version 1 represents the first draft. Changes were made to the statistical design, since we agreed on the hypothesis that a drainage period of 6 and/or 12 h is non-inferior to 24 h of drainage regarding recurrence rate. Therefore, we moved away from a “superiority approach” to a “non-inferiority” multi-arm, multi-stage (MAMS) design, which enables adaptive reductions of the number of experimental arms during the trial. No further changes were made.

## Supplementary Information

**Additional file 1.** Supplementary A.

## Abbreviations

CSDH: Chronic subdural hematoma
REDCap: Research Electronic Data Capture system
DACSUHS: Danish Chronic Subdural Hematoma Study group
CT: Computed tomography
MRI: Magnetic resonance imaging
mRS: Modified Rankin Scale
SF-36: Short Form-36
HRQOL: Health-related quality of life
GCS: Glasgow Coma Scale
OPEN: Open Patient data Explorative Network
GCP: Good Clinical Practice
eCRF: Electronic case report form
FDA: Food and Drug Administration
OR: Odds ratio
RD: Risk difference

## References

[1] Santarius T, Kirkpatrick PJ, Ganesan D (2009). Use of drains versus no drains after burr-hole evacuation of chronic subdural haematoma: a randomised controlled trial. Lancet.

[2] Jensen TSR, Andersen-Ranberg N, Poulsen FR (2020). The Danish Chronic Subdural Hematoma Study-comparison of hematoma age to the radiological appearance at time of diagnosis. Acta Neurochir.

[3] Glancz LJ, Poon MTC, Coulter IC (2019). Does drain position and duration influence outcomes in patients undergoing burr-hole evacuation of chronic subdural hematoma? Lessons from a UK multicenter prospective cohort study. Neurosurgery.

[4] Peng D, Zhu Y. External drains versus no drains after burr-hole evacuation for the treatment of chronic subdural haematoma in adults. Cochrane Database Syst Rev. 2016:CD011402. 10.1002/14651858.CD011402.pub2. 10.1002/14651858.CD011402.pub2 PMC7083261 27578263

[5] Ibrahim I, Maarrawi J, Jouanneau E (2010). Evacuation of chronic subdural hematomas with the twist-drill technique: results of a randomized prospective study comparing 48-h and 96-h drainage duration. Neurochirurgie.

[6] Bratton DJ, Phillips PP, Parmar MK (2013). A multi-arm multi-stage clinical trial design for binary outcomes with application to tuberculosis. BMC Med Res Methodol.

[7] Munetz MR, Lidz CW, Meisel A (1985). Informed consent and incompetent medical patients. J Fam Pract.

[8] Thorbjørn SRJ, Nina AR, Frantz RP, Bo B, Kåre F. Behandling af kronisk subduralt hæmatom. In: Ugeskrift for læger. https://ugeskriftet.dk/files/scientific_article_files/2018-10/v03180160.pdf. Accessed 1 May 2021.

[9] Althunian TA, de Boer A, Klungel OH, et al. Methods of defining the non-inferiority margin in randomized, double-blind controlled trials: a systematic review. Trials. 2017;18:–107. 10.1186/s13063-017-1859-x. 10.1186/s13063-017-1859-x PMC5341347 28270184

[10] U.S. Department of Health and Human Services, Food and Drug Administration, Center for Drug Evaluation and Research (CDER), Center for Biologics Evaluation and Research (CBER) (2010). Guidance for industry, non-inferiority clinical trials.

[11] Liu W, Bakker NA, Groen RJ (2014). Chronic subdural hematoma: a systematic review and meta-analysis of surgical procedures. J Neurosurg.

[12] NSTAGEBIN: Stata module to perform sample size calculation for multi-arm multi-stage randomised controlled trials with binary outcomes. Statistical Software Components S457911, Boston College Department of Economics, revised 24 Sep 2014. https://ideas.repec.org/c/boc/bocode/s457911.html. Accessed 1 June 2021.

[13] R Core Team. R: a language and environment for statistical computing. R Foundation for Statistical Computing, Vienna, Austria. 2021. Https://www.R-project.org/. Accessed 1 May 2021.

[14] Robitzsch A, Grund S. Miceadds: some additional multiple imputation functions, especially for ‘mice’. R. package version 3.11-6. 2021. Https://CRAN.R-project.org/package=miceadds. Accessed 1 May 2021.

[15] Banks JL, Marotta CA (2007). Outcomes validity and reliability of the modified Rankin scale: implications for stroke clinical trials: a literature review and synthesis. Stroke.

[16] Broderick JP, Adeoye O, Elm J (2017). Evolution of the Modified Rankin Scale and its use in future stroke trials. Stroke.

[17] Harrison JK, McArthur KS, Quinn TJ (2013). Assessment scales in stroke: clinimetric and clinical considerations. Clin Interv Aging.

[18] Quinn TJ, Dawson J, Walters MR (2009). Reliability of the Modified Rankin Scale: a systematic review. Stroke.

[19] Kalpakjian CZ, Forchheimer M, Tate DG, Sisto SA, Druin E, Sliwinski MM (2009). Chapter twenty-three - Quality of life after spinal cord injury. Spinal cord injuries.

[20] Tully T, Patel AS, Birring SS, Baughman RP, Valeyre D (2019). Chapter 18 - Quality of life. Sarcoidosis.

[21] Von Der Heyde R, Cooper C (2007). Chapter 6 - Assessment of functional outcomes. Fundamentals of hand therapy.

[22] Pedersen CB, Sundbye F, Poulsen FR (2017). No value of routine brain computed tomography 6 weeks after evacuation of chronic subdural hematoma. Surg J (N Y).

[23] Eurostat. Stastics explained. https://ec.europa.eu/eurostat/statistics-explained/index.php?title=Population_structure_and_ageing. Accessed 1 July 2021.

