# Supplementary material for: Postoperative drainage for 6, 12, or 24 h after burr-hole evacuation of chronic subdural hematoma in symptomatic patients (DRAIN-TIME 2): study protocol for a nationwide randomized controlled trial
Source: Trials. 2022 Mar 14;23:213. doi: 10.1186/s13063-022-06150-x (PMC8919549; doi:10.1186/s13063-022-06150-x)
Supplement: Supplementary file 1 — Additional file 1. Supplementary A. [file 13063_2022_6150_MOESM1_ESM.docx]

**Supplementary material A**

. nstagebin, accrate(300 300 300 300 300) arms(3 3 3 3 3) theta0(0.07) theta1(0) ctrlp(0.837) alpha(0.5 .3 0.2 .1 .025) power(0.9 0.9

> .95 .95 .95) fu(0) ltfu(0) aratio(1) extrat(0) nstage(5) tunit(1) ess probs

n-stage trial design version 1.0.1, 17 Jul 2014

---------------------------------------------------------------

Sample size for a 3-arm 5-stage trial with binary outcome based

on Bratton et al. (2013) BMC Med Res Meth 13:139

---------------------------------------------------------------

Control arm event rate = 0.84

Operating characteristics

----------------------------------------------------------------------

Alpha(1S) Power theta|H0 theta|H1 Length* Time*

----------------------------------------------------------------------

Stage 1 0.5000 0.900 0.070 0.000 0.910 0.910

Stage 2 0.3000 0.900 0.070 0.000 0.910 1.820

Stage 3 0.2000 0.950 0.070 0.000 1.620 3.440

Stage 4 0.1000 0.950 0.070 0.000 1.330 4.770

Stage 5 0.0250 0.950 0.070 0.000 2.470 7.240

Pairwise 0.0148 0.802 7.240

FWER (SE) 0.0277 (0.0003)

----------------------------------------------------------------------

* Length (duration of each stage) is expressed in year periods

Cumulative sample sizes per arm per stage

---------Stage 1--------- ---------Stage 2---------

Overall Control Exper. Overall Control Exper.

---------------------------------------------------------------------------

Number of active arms 3 1 2 3 1 2

Accrual rate* 300.0 100.0 200.0 300.0 100.0 200.0

Active arms

Patients for analysis 273 91 91 546 182 182

Patients recruited** 273 91 91 546 182 182

All arms

Patients recruited** 273 546

---------------------------------------------------------------------------

---------Stage 3--------- ---------Stage 4---------

Overall Control Exper. Overall Control Exper.

---------------------------------------------------------------------------

Number of active arms 3 1 2 3 1 2

Accrual rate* 300.0 100.0 200.0 300.0 100.0 200.0

Active arms

Patients for analysis 1032 344 344 1431 477 477

Patients recruited** 1032 344 344 1431 477 477

All arms

Patients recruited** 1032 1431

---------------------------------------------------------------------------

---------Stage 5---------

Overall Control Exper.

------------------------------------------------

Number of active arms 3 1 2

Accrual rate* 300.0 100.0 200.0

Active arms

Patients for analysis 2172 724 724

Patients recruited** 2172 724 724

All arms

Patients recruited** 2172

------------------------------------------------

* Accrual rates are specified in number of patients per year

** Accounts for loss-to-follow-up rate and includes those recruited during follow-up periods

Expected sample size | 0 effective arms = 690

Expected sample size | 2 effective arms = 1928

Prob. of k experimental arms passing each stage: 0 effective arms

------------------------------------

k(# arms) 0 1 2

------------------------------------

Stage 1 0.332 0.334 0.334

Stage 2 0.611 0.267 0.122

Stage 3 0.785 0.169 0.045

Stage 4 0.892 0.092 0.016

Stage 5 0.972 0.026 0.002

------------------------------------

Prob. of k experimental arms passing each stage: 2 effective arms

------------------------------------

k(# arms) 0 1 2

------------------------------------

Stage 1 0.033 0.136 0.832

Stage 2 0.057 0.189 0.754

Stage 3 0.066 0.205 0.729

Stage 4 0.073 0.218 0.709

Stage 5 0.081 0.232 0.687
